# Prediction of antimicrobial peptides toxicity based on their physico-chemical properties using machine learning techniques

**DOI:** 10.1186/s12859-021-04468-y

**Published:** 2021-11-10

**Authors:** Hossein Khabbaz, Mohammad Hossein Karimi-Jafari, Ali Akbar Saboury, Bagher BabaAli

**Affiliations:** 1 Institute of Biochemistry and Biophysics, University of Tehran, Tehran, Iran; 2 School of Mathematics, Statistics and Computer Science, College of Science, University of Tehran, Tehran, Iran

**Keywords:** Antimicrobial peptides, Peptide toxicity, Machine learning, Physico-chemical properties

## Abstract

**Background:**

Antimicrobial peptides are promising tools to fight against ever-growing antibiotic resistance. However, despite many advantages, their toxicity to mammalian cells is a critical obstacle in clinical application and needs to be addressed.

**Results:**

In this study, by using an up-to-date dataset, a machine learning model has been trained successfully to predict the toxicity of antimicrobial peptides. The comprehensive set of features of both physico-chemical and linguistic-based with local and global essences have undergone feature selection to identify key properties behind toxicity of antimicrobial peptides. After feature selection, the hybrid model showed the best performance with a recall of 0. 876 and a F1 score of 0. 849.

**Conclusions:**

The obtained model can be useful in extracting AMPs with low toxicity from AMP libraries in clinical applications. On the other hand, several properties with local nature including positions of strand forming and hydrophobic residues in final selected features show that these properties are critical definer of peptide properties and should be considered in developing models for activity prediction of peptides. The executable code is available at https://git.io/JRZaT.

**Supplementary Information:**

The online version contains supplementary material available at 10.1186/s12859-021-04468-y.

## Background

Antimicrobial peptides (AMPs) are olden defensive tools of living organisms against microbial infections [1]. AMPs are highly diverse in terms of length, sequence and their structure. This diversity suggests that these peptides have wide range of mechanisms of action on targets [2]. AMPs have many advantages compared to traditional antibiotics such as broad-spectrum antimicrobial activity, selectivity for bacterial cells and rare occurrence of resistance [3]. Despite these benefits, toxicity of AMPs to mammalian cells is still the main concern in developing AMPs and is a major obstacle in their clinical applications [4].

Experimental identification and development of new antimicrobial peptides is both highly expensive and time-consuming. Hence, development of computational models is essential to provide the fast analysis of potential AMP candidates by predicting their activities before their synthesis. Besides that, machine learning techniques can be used to determine the crucial physicochemical properties behind AMPs biological functionalities [5].

Recently, many studies have been dedicated to develop predicting models using machine learning techniques to classify AMP candidates based on their sequences [6–10]. In 2016, Chaudhary et al. [7] developed a tool to predict hemolytic activity of peptides. Features used in their work were mostly linguistic-based and involved physicochemical properties with global nature. Also, the classification performance for discriminating highly hemolytic and poorly hemolytic peptides has still room to improve. In another study by Kleandrova et al. [4], simultaneous prediction of antibacterial activity and cytotoxicity with high accuracy was carried out using a limited set of features of Broto-Moreau autocorrelations [11].

**Fig. 1:**
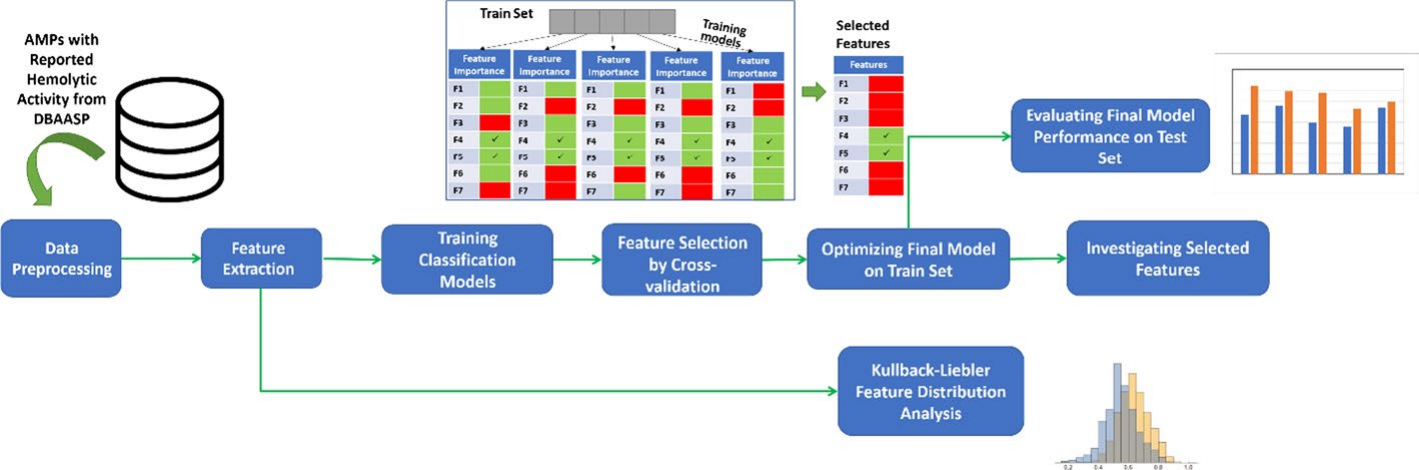
General steps taken for development of toxicity prediction model and investigating involved peptide properties

In this work, we aim to take on both of these issues by training a model with an inclusive set of features on an up-to-date dataset for prediction of toxicity of AMPs. General steps taken here are illustrated in Fig. 1. Features set include both physico-chemical and linguistic-based properties with both local and global nature. Most important properties behind the toxicity are also investigated by implementing feature selection by cross-validation and distribution distant analysis of toxic and non-toxic AMPs.

## Results and discussion

### Preparation of dataset for training models

Several AMP databases were considered to obtain data from, yet the DBAASP dataset was chosen. This database is equipped with application programmable interface and it is still being updated while most other databases were outdated. DBAASP provides access to latest experimental data of AMPs antimicrobial activity and toxicity. Multiple considerations were necessary before using this dataset. Since property calculation algorithms mostly recognize natural amino acids, many AMP records with unnatural residues or D-amino acids and terminal modification other than amid and acetyl group were removed. All concentration values with “µg/ml” unit were converted to “µM” using the molecular weights of peptides. This conversion was necessary to be able to compare AMPs and also label them according to labeling rules. Several types of toxicity values including HC50, CC50 and MIC were available in data. The transformations of the HC50, CC50 and MIC toxicity values allowed for a more accurate comparison and labeling since it’s based on peptide activity concentration. After these processes, dataset was ready for labeling and property calculation.

### Analysis of distributions of features among toxic and non-toxic AMPs

**Fig. 2:**
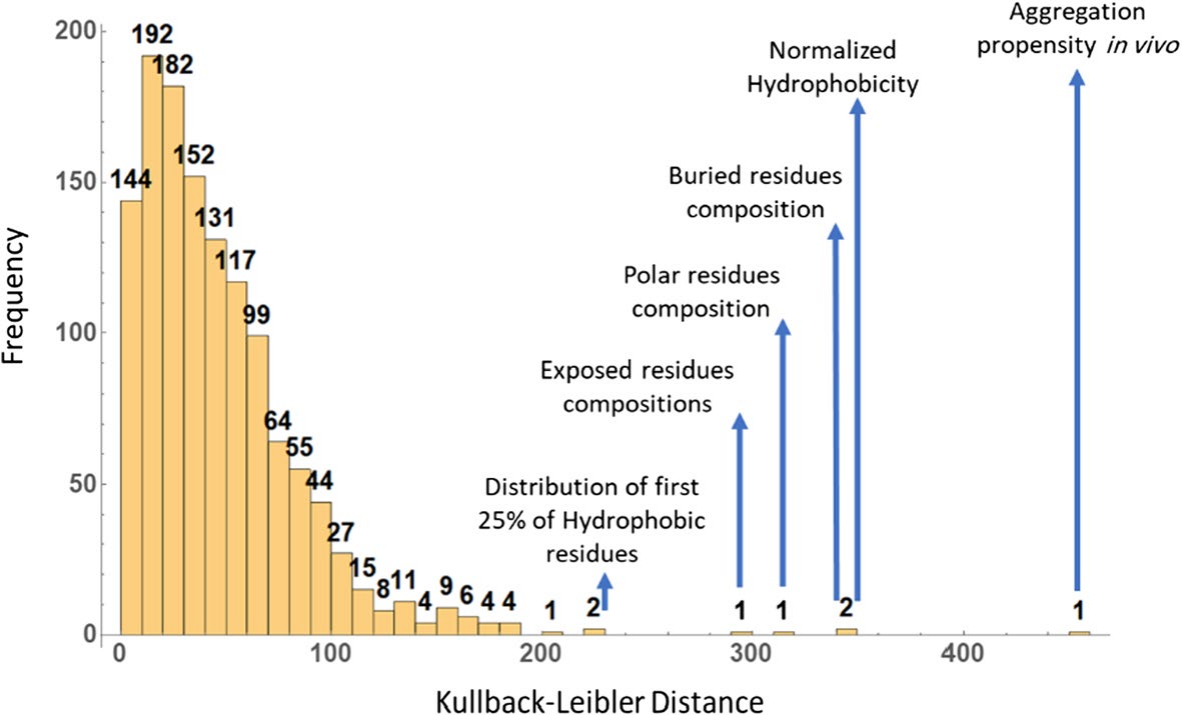
Histogram of Kullback–Liebler distance values calculated for each feature between the two sets of toxic and non-toxic AMPs

After calculating all features and removing correlated features, it was interesting to see if distinctive features could be found by comparing distributions of features among toxic and non-toxic AMPs. It should be noted that, all records in the dataset are AMPs and therefore they certainly have much in common in terms of features in basically all categories. Results from applying T-distributed Stochastic Neighbor Embedding (t-SNE) on final dataset (Additional file 1: Fig. S1) also shows that toxic and non-toxic AMPs have very similar feature distributions. Using Kullback–Leibler divergence method, comparison of toxic and non-toxic distributions for all 1263 features were carried out. As can be seen in the Fig. 2, although in most cases, the difference between feature distributions among toxic and non-toxic AMPs are negligible, some properties including Aggregation propensity in vivo, Normalized hydrophobicity and Composition of buried residues show distinct distributions.

### Training model for classification of toxic and non-toxic AMPs

**Fig. 3:**
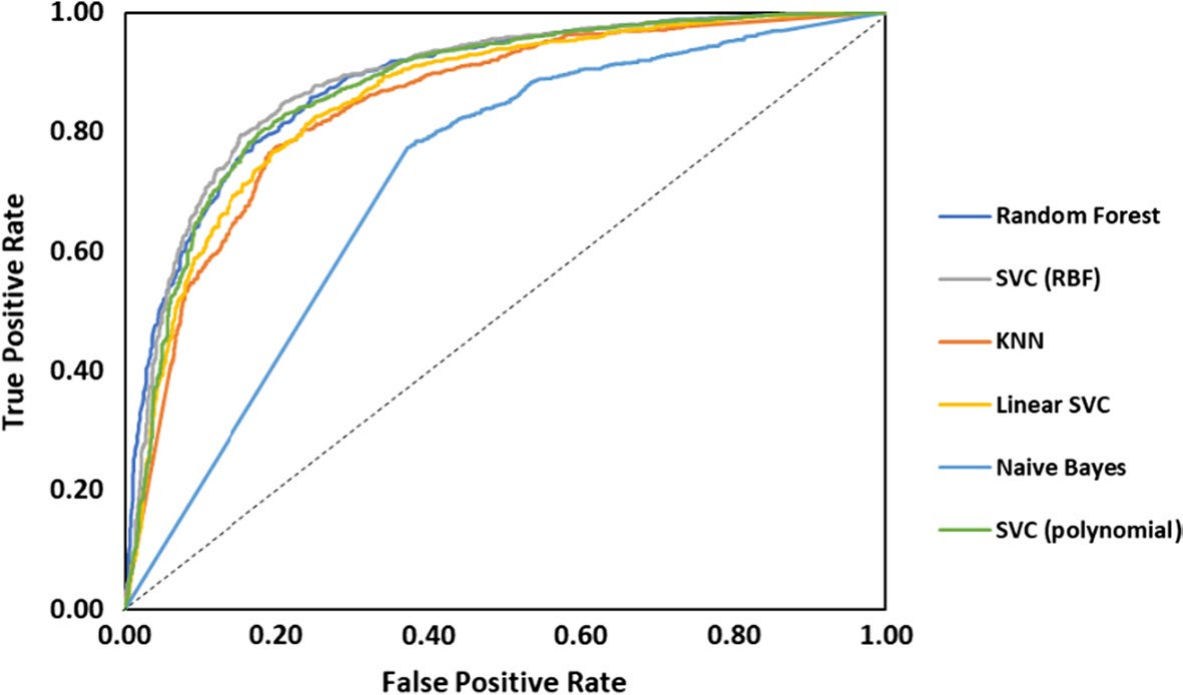
Comparison of ROC curves of different algorithms before feature selection

In order to achieve a model for discriminating toxic and non-toxic AMPs, three steps of dimension reduction were carried out along the way. After each step, SVC, Linear SVC, Random Forest, KNN and hybrid models were trained and optimized on the training set. Receiver operating characteristic (ROC) curves were used to determine and compare the performances of the models. The Area Under the Curve (AUC) is used for measuring the ability of a classifier to distinguish between classes. The higher the AUC, the better the performance. Figure 3 shows the ROC results before performing feature selection. The dotted line shows the performance of a completely random classifier (AUC = 0.5). As can be seen in the figure and Table 1, Random Forest and SVC model show comparable results and have achieved higher AUC scores.

**Table 1:** Comparison of area under curves of different algorithms before feature selection

Algorithm	AUC before feature selection
Random Forest	0.8835
SVC (RBF)	0.8862
SVC (Polynomial)	0.8752
Naïve Bayes	0.7165
Linear SVC	0.8575
KNN	0.8452

**Fig. 4:**
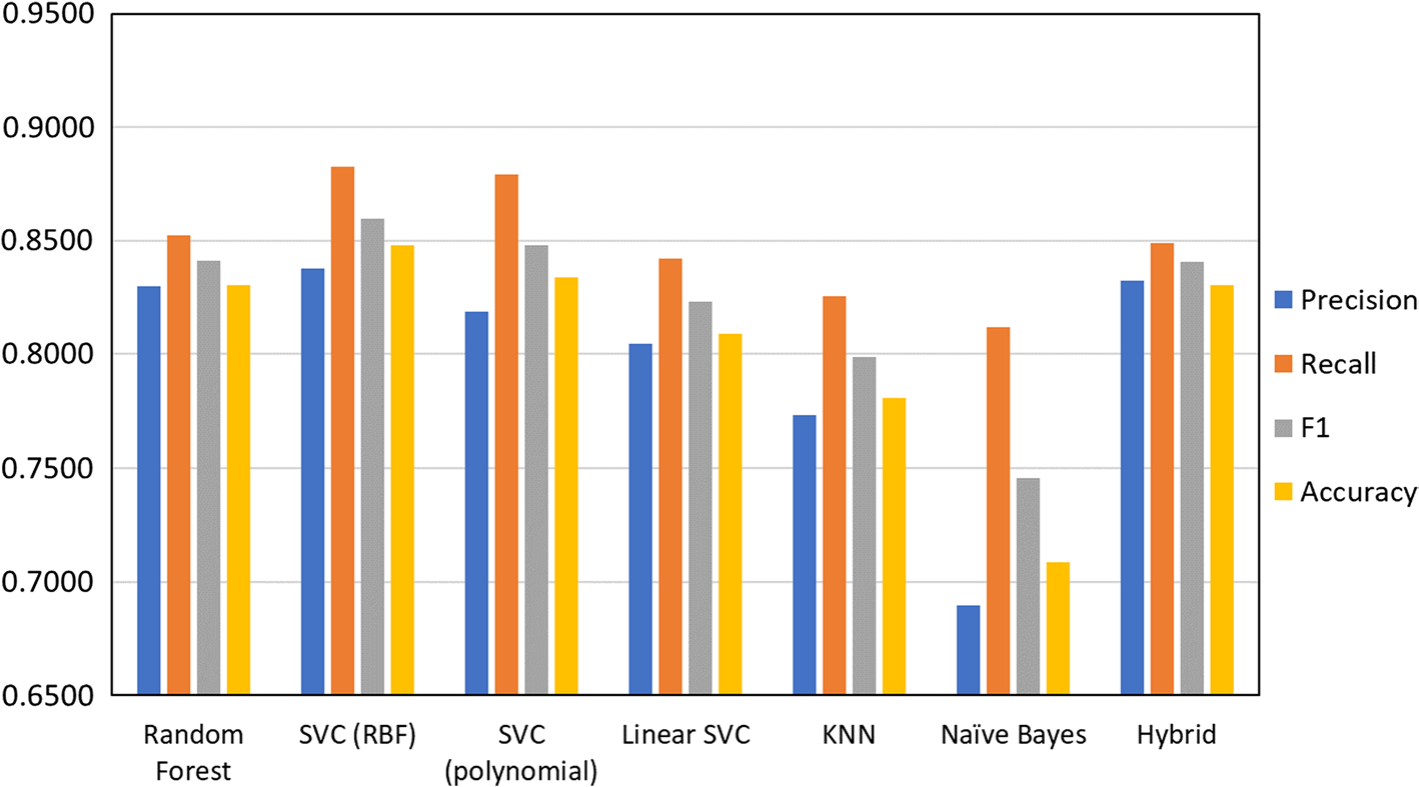
Comparison of different algorithms results in classification performance measures before feature selection

In this scenario, it was important to minimize the number of false-non-toxic predictions while also preventing the model to predict all samples as toxic. Therefore, F1 score was chosen as the performance measure to be optimized since it manages to consider both Recall (to minimize false-non-toxic) and Precision (to maximize true-non-toxic). Figure 4 compares these key performance measures of all implemented algorithms. Here, SVC (RBF, c = 5, gamma = 0.03), SVC (Polynomial, c = 0.001, gamma = 0.2), Random forest and hybrid models show better results compared to that of Linear SVC (c = 0.2), Naïve Bayes and KNN (k = 5). Considering these results, Linear SVC, Naïve Bayes and KNN algorithms were omitted in the following steps. Compared to work of Chaudhary et al. [7] here we were able to achieve a roughly 9% increase in the accuracy of model.

### Feature selection and model performances

Here, obtaining a model to discriminate toxic and non-toxic AMPs is not our only goal. We also want to investigate the underlying properties responsible for this difference in toxicity. These properties can either be based on amino acid sequence of peptide or have physico-chemical nature. Many works have been published which used sequence-based properties and successfully predicted a peptide function [7, 8]. However, performances of these models depend highly on the similarity of query peptide sequence with the indexed peptides in the database. Considering that only a very small fraction of sequence space has enough similarity to known AMPs (even without considering the peptide length), these models will have arguable performance in many high-throughput applications such as peptide design.

Ideally, a model based on exact physico-chemical properties responsible for the toxicity of peptide should perform sufficiently good. However, that would be the case in our work only if the extracted properties cover all of those essential properties. Although in many works [12–15] the underlying properties behind toxicity of AMPs have been investigated, there’s still much to understand about this phenomenon. Accordingly, in order to cover as much properties as we could, we used Propy package to calculate peptide physico-chemical properties. On the other hand, since considered physico-chemical properties may not have enough information about the AMP activity, sequence-based descriptors were also calculated to have a comprehensive set of features and let the model choose the most informative descriptors in feature selection steps.

**Fig. 5:**
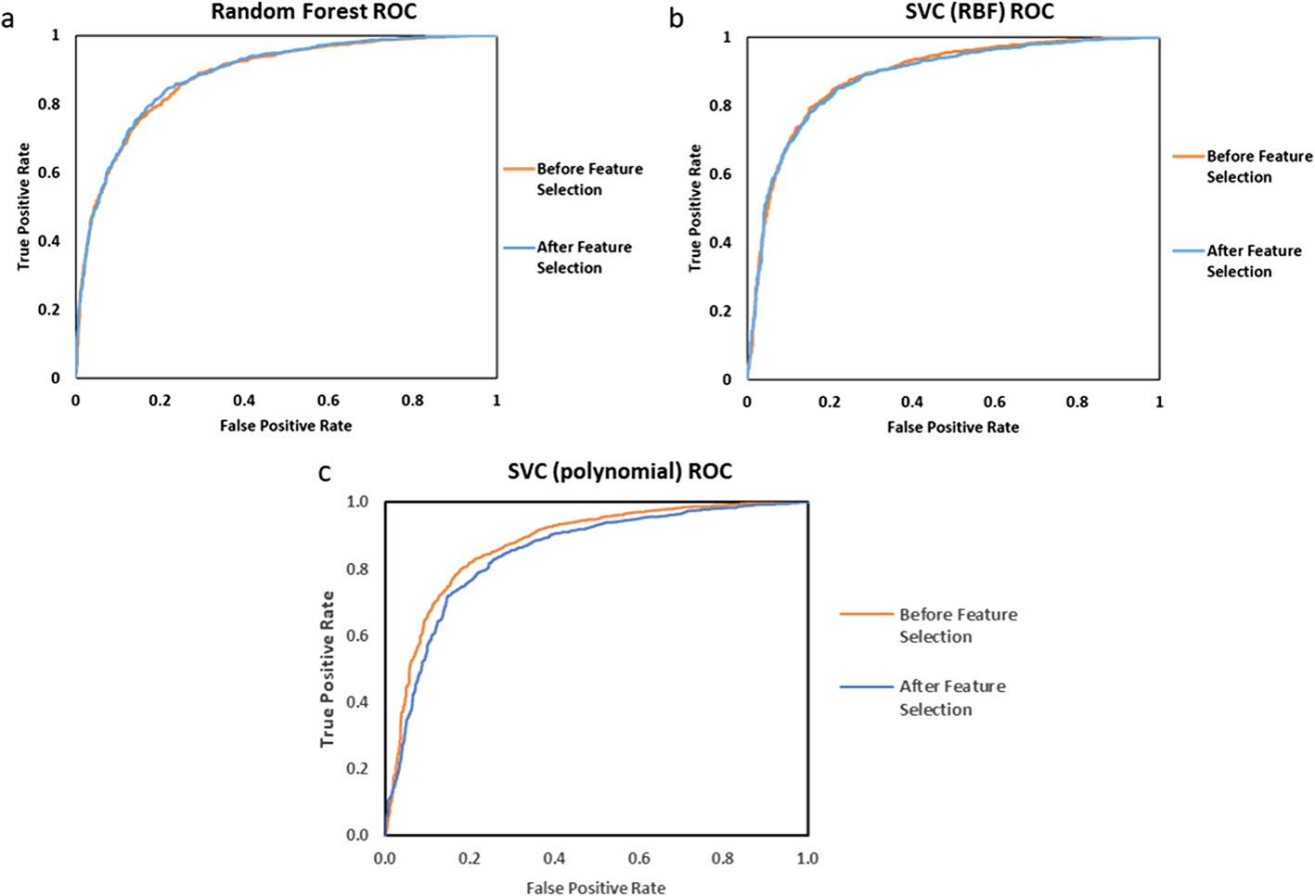
ROC performance of Random Forest (**a**) SVC (RBF) model (**b**) and SVC (polynomial) model (**c**) after feature selection

In order to get to an interpretable number of features, after removing correlated features, two methods of feature selection including L1-SVM and Tree-Based feature selection by cross validation were carried out sequentially. After these steps, 1276 features were down to 90 features (Additional file 4: Table S3, Additional file 5: Table S4). Figure 5 shows the ROC results of random forest (a), SVC (RBF, c = 7, gamma = 0.35) (b) and SVC (Polynomial, c = 0.1, gamma = 0.55) (c) models and corresponding AUCs have been shown in Table 2. No significant performance losses were obtained after performing feature selection except for SVC (polynomial). Here, random forest model had the highest AUC.

**Table 2:** Comparison of AUC performances of random forest and SVC models before and after feature selection

Algorithm	AUC before feature selection	AUC after feature selection
Random Forest	0.8835	0.8856
SVC (RBF)	0.8862	0.8814
SVC (Polynomial)	0.8752	0.8477

**Fig. 6:**
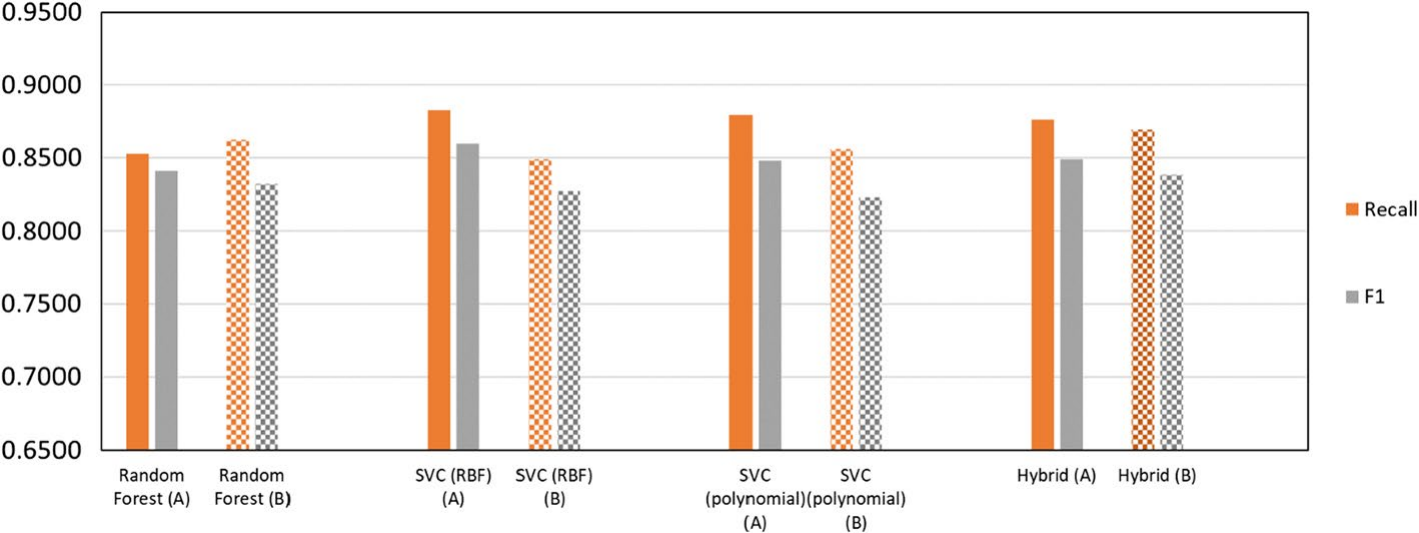
Comparison of classification results before (A) and after (B) feature selection

Using hamming distance, performances of different algorithms were also compared with the hybrid model and show differences between 0.0247 and 0.0883 (Additional file 2: Table S1). Results on test set from final models are shown in Fig. 6. As can be seen in the figure, although SVC (RBF) shows higher performance before feature selection, the hybrid model acts better with selected features in terms of F1 Score which shows that combination of various algorithms has helped here too achieve higher performance. Considering the application of this model, it favors us to have a model with lowest chance of predicting a false non-toxic AMP while still being able to detect non-toxic AMPs. As for the performance measures, this preference is reflexed mostly in Recall and F1 Score which have been obtained 0.849and 0.849, respectively. The hybrid model showed no significant performance loss with selected features. Therefore, it can be concluded that these features, collectively, have essential information for prediction of toxicity of an AMP.

**Fig. 7:**
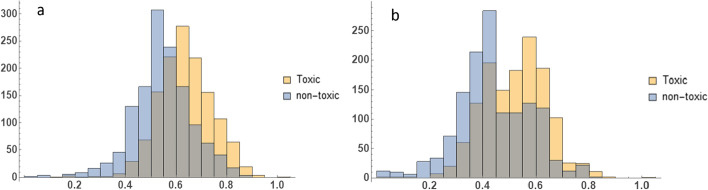
Comparison of distributions of calculated in vivo aggregation propensity (**a**) and polar residue composition (**b**) among toxic and non-toxic AMPs

Using feature importance attribute of random forest model, it was discovered that aggregation propensity of peptide in vivo is the most distinctive feature for toxicity of peptide. The algorithm behind calculating this feature can predict the peptide aggregation propensity in the presence of cell material [16]. Interestingly, in the Kullback–Leibler distance results, this feature has the highest value which shows that it has the most distant distributions in toxic and non-toxic AMPs (Fig. 7a). The composition of polar residues ranked second in final 90 features. By definition, it’s the number of polar residues (including Leu, Ile, Phe, Trp, Cys, Met, Val and Tyr) divided by total number of residues in the peptide. Similarly, it was one of the top 5 features in Kullback–Leibler distance analysis (Fig. 7b).

A full list of selected features has been shown and categorized in Additional file 3: Tables S2 and Additional file 4: Table S3. Most of these features were based on physicochemical properties of AMPs including charge, hydrophobicity, polarity, secondary structure and solvent accessibility. Many of these attributes are based on distribution of properties and are independent of the amino acid composition which demonstrates the importance of properties with local nature. The fractional distance along the peptide sequence that must be traveled to encounter the first residue with high strand forming propensity (VIYCWFT) and the first hydrophobic residue (CLVIMFW) are among properties with local nature. There are also some properties which measures fraction of pairs of contiguous residues that belong to each of the possible combinations of different categories. For instance, fraction of pairs of contiguous residues with considerable difference in polarity and hydrophobicity value are among the top important properties. AMPs with similar amino acid compositions can have different values for these properties based on the distribution of residues. This result confirms that features only with a global nature such as the ones obtained from the sequences of the AMPs are not informative enough to predict the activity of a peptide.

## Conclusion

Here, by using an up-to-date dataset, we developed a machine learning model to predict the toxicity of antimicrobial peptides with excellent performance. Feature selection by cross validation was carried out on an inclusive set of features of both physico-chemical and linguistic-based to identify crucial features involved in the toxicity of antimicrobial peptides. It has also been shown that local properties have crucial role in peptide functionality and therefore need to be considered in training new models. This model can be used as a tool for extracting AMPs with low toxicity from AMP libraries.

## Methods

### Preparing data

All AMP records were collected from Database of Antimicrobial Activity and Structure of Peptides (DBAASP) [17]. Records with reported quantitative hemolytic activity have been imported from the database. AMPs with unnatural residues (unusual amino acids, D-amino acids), C-terminal modifications other than amid group and N-terminal modifications other than acetyl group have been removed. In addition, peptides shorter than 6 residues and longer than 50 residues were also removed from dataset due to lack of sufficient examples in those length ranges. Concentration values with μg/mL were divided by the molecular weight of the corresponding peptide and then multiplied by 1000, to have all the values in μM unit. The initial range of AMPs concentrations in their hemolytic activity was shortened to the range of 0.5–1000 µM. In addition, in the final dataset, peptides with no reported activity against lipid bilayer were also eliminated.

Using peptides concentration and lysis values and based on Additional file 5: Table S4, “Toxic” and “Non-toxic” labels were assigned to each record to prepare the data for training classification models. Most of AMPs had more than 1 reported hemolytic activity, so these peptides potentially could have different labels. The final dataset included only peptides with a single label of toxic or non-toxic. For example, if a peptide turns out to be toxic in one hemolytic report, and non-toxic in another report, all records of this peptide were removed from the dataset.

### Feature extraction

Here a total number of 1541 features have been extracted from peptide sequences (Additional file 6: Table S5). Freely-available Propy python package [18] was used to extract 1527 features from categories including amino acid composition, dipeptide composition, autocorrelation, pseudo-amino acid composition and sequence order properties. Each record in the obtained data from DBAASP already had four physico-chemical properties of normalized hydrophobic moment, normalized hydrophobicity, net charge and isoelectric point. The disordering property and charge density were calculated similar to a previous work [9]. Aggregation propensity in vitro and in vivo were calculated using AGGRESCAN web server [16] and TANGO software [19], respectively. The Mean Hydrophilicity, Steric Hinderance, Solvation, Hydropathy and Amphiphilicity were calculated using data from AAIndex database [20] (Additional file 7).

### Training machine learning models

Here, several models including Support Vector Classifier (SVC) with radial basis function (RBF) and Polynomial kernels, Linear Support Vector Classifier (LSVC), Random Forest, Naïve Bayes and K-Nearest Neighbor were trained to predict the toxicity of AMPs. A voting classifier which is a hybrid model of all these algorithms was also trained. The train set (80%) and test set (20%) were constructed with no overlap between them and with enabled stratify argument on the class value (toxic, or non-toxic). All trainings have been carried out using Scikit-learn [21] Python library with tenfold cross validation on train set. All models were optimized on train set using grid search by cross-validation and then the best model was used on test set. Comparison of model performances was carried out using performance measures (including precision, recall, f1-score, accuracy and AUC) and hamming distance.

### Feature selection strategy

In order to remove redundant or highly correlated features, several feature selection methods have been implemented. Pearson correlations between all pairs of features have been calculated using Mathematica software [22]. Tree-based feature selection and L1-based feature selection have been carried out separately using Scikit-Learn [21] Python library. First, the input data has been randomly split into 5 folds. The classifier (Random Forest classifier or Linear Support Vector Classifier) has been trained on each part. Lastly, in each method, features which were shared among all 5 folds, have been extracted for further usage.

### Kullback–Leibler distance

To compare distributions of toxic and non-toxic AMPs on each feature, Kullback–Leibler (KL) distance [23] of distributions for all features were calculated and compared. Kullback–Leibler divergence quantifies the difference of two distributions for a given variable. Here, KL Divergence was measured for all calculated features independent of the feature selection steps. KL Divergence is calculated as follow:

$${\varvec{D}}_{{{\varvec{KL}}}} ({\varvec{p}}|{|}{\varvec{q}}{)} = \mathop \sum \limits_{{{\varvec{i}} = 1}}^{{\varvec{N}}} {\varvec{p}}\left( {{\varvec{x}}_{{\varvec{i}}} } \right) \cdot {\varvec{log}}\frac{{{\varvec{p}}\left( {{\varvec{x}}_{{\varvec{i}}} } \right)}}{{{\varvec{q}}\left( {{\varvec{x}}_{{\varvec{i}}} } \right)}}$$

where p and q are two probability distributions of variable x_i_. To obtain KL distance, KL Divergence was calculated twice with interchanged values of p and q and the mean value for each variable was reported as KL distance.

### Visualization of feature distribution

Using t-SNE method in Scikit-learn package, the high dimensional space of AMP features (90) was brought to 2 components to visualize and compare the feature distribution among toxic and non-toxic AMPs.

## Supplementary Information

**Additional file 1: Figure S1.** Applying t-SNE on final dataset and reducing dimensions from 90 to 2.

**Additional file 2: Table S1.** Hamming distance between hybrid and other models.

**Additional file 3: Table S2.** Distribution of final features in each feature category and sub-category.

**Additional file 4: Table S3.** Final features sorted by their importance.

**Additional file 5: Table S4.** Labeling rules for toxic and non-toxic AMPs.

**Additional file 6: Table S5.** Feature categories calculated for each peptide.

**Additional file 7.** AMP-Dataset.zip. The dataset used here for training models.

## Data Availability

The dataset supporting the conclusions of this article is included within the article (AMP-Dataset.zip). The execution code is available on Github at https://git.io/JRZaT.

## Abbreviations

AMP: Antimicrobial peptides
SVC: Support vector Classifier
LSVC: Linear Support Vector Classifier
DBAASP: Database of Antimicrobial Activity and Structure of Peptides
AUC: Area Under the Curve
ROC: Receiver operating characteristic
KL: Kullback-Leibler
t-SNE: T-distributed Stochastic Neighbor Embedding
RBF: Radial Basis Function

## References

[1] Zasloff M (2002). Antimicrobial peptides in health and disease. N Engl J Med.

[2] Lee TH, Hall KN, Aguilar MI (2016). Antimicrobial peptide structure and mechanism of action: a focus on the role of membrane structure. Curr Top Med Chem.

[3] Haney EF, Hancock RE (2013). Peptide design for antimicrobial and immunomodulatory applications. Biopolymers.

[4] Kleandrova VV, Ruso JM, Speck-Planche A, Dias Soeiro Cordeiro MN (2016). Enabling the discovery and virtual screening of potent and safe antimicrobial peptides. Simultaneous prediction of antibacterial activity and cytotoxicity. ACS Comb Sci.

[5] Lee EY, Lee MW, Fulan BM, Ferguson AL, Wong GC (2017). What can machine learning do for antimicrobial peptides, and what can antimicrobial peptides do for machine learning?. Interface Focus.

[6] Lee EY, Fulan BM, Wong GC, Ferguson AL (2016). Mapping membrane activity in undiscovered peptide sequence space using machine learning. Proc Natl Acad Sci.

[7] Chaudhary K, Kumar R, Singh S, Tuknait A, Gautam A, Mathur D (2016). A web server and mobile app for computing hemolytic potency of peptides. Sci Rep.

[8] Gupta S, Kapoor P, Chaudhary K, Gautam A, Kumar R, Raghava GP (2013). In silico approach for predicting toxicity of peptides and proteins. PLoS ONE.

[9] Vishnepolsky B, Pirtskhalava M (2014). Prediction of linear cationic antimicrobial peptides based on characteristics responsible for their interaction with the membranes. J Chem Inf Model.

[10] Su X, Xu J, Yin Y, Quan X, Zhang H (2019). Antimicrobial peptide identification using multi-scale convolutional network. BMC Bioinform.

[11] Todeschini R, Consonni V (2008). Handbook of molecular descriptors.

[12] Oren Z, Shai Y (1997). Selective lysis of bacteria but not mammalian cells by diastereomers of melittin: structure−function study. Biochemistry.

[13] Sitaram N, Nagaraj R (1999). Interaction of antimicrobial peptides with biological and model membranes: structural and charge requirements for activity. Biochim Biophys Acta (BBA) Biomembranes.

[14] Dathe M, Nikolenko H, Meyer J, Beyermann M, Bienert M (2001). Optimization of the antimicrobial activity of magainin peptides by modification of charge. FEBS Lett.

[15] Chou H-T, Kuo T-Y, Chiang J-C, Pei M-J, Yang W-T, Yu H-C (2008). Design and synthesis of cationic antimicrobial peptides with improved activity and selectivity against Vibrio spp. Int J Antimicrob Agents.

[16] de Groot NS, Castillo V, Graña-Montes R, Ventura S (2012). AGGRESCAN: method, application, and perspectives for drug design. Computational drug discovery and design.

[17] Gogoladze G, Grigolava M, Vishnepolsky B, Chubinidze M, Duroux P, Lefranc M-P (2014). DBAASP: database of antimicrobial activity and structure of peptides. FEMS Microbiol Lett.

[18] Cao D-S, Xu Q-S, Liang Y-Z (2013). propy: a tool to generate various modes of Chou’s PseAAC. Bioinformatics.

[19] Fernandez-Escamilla A-M, Rousseau F, Schymkowitz J, Serrano L (2004). Prediction of sequence-dependent and mutational effects on the aggregation of peptides and proteins. Nat Biotechnol.

[20] Kawashima S, Kanehisa M (2000). AAindex: amino acid index database. Nucleic Acids Res.

[21] Pedregosa F, Varoquaux G, Gramfort A, Michel V, Thirion B, Grisel O (2011). Scikit-learn: machine learning in Python. J Mach Learn Res.

[22] Wolfram Research I. Mathematica. Champaign, Illinois; 2020.

[23] Kullback S (1997). Information theory and statistics.

