# Supplementary figures and images for: Prediction of antimicrobial peptides toxicity based on their physico-chemical properties using machine learning techniques
Source: BMC Bioinformatics. 2021 Nov 10;22:549. doi: 10.1186/s12859-021-04468-y (PMC8582201; doi:10.1186/s12859-021-04468-y)

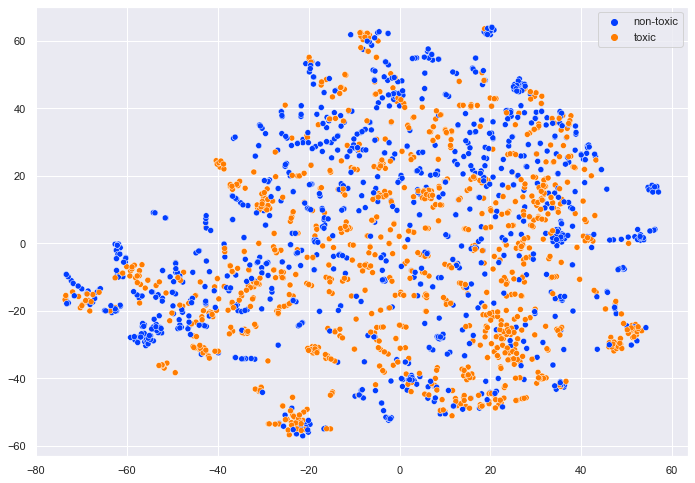


Figure S1. Applying t-SNE on final dataset and reducing dimensions from 90 to 2.

Supplement: Supplementary file 1 — Additional file 1: Figure S1. Applying t-SNE on final dataset and reducing dimensions from 90 to 2. [file 12859_2021_4468_MOESM1_ESM.docx]
