# Supplementary material for: Prediction of antimicrobial peptides toxicity based on their physico-chemical properties using machine learning techniques
Source: BMC Bioinformatics. 2021 Nov 10;22:549. doi: 10.1186/s12859-021-04468-y (PMC8582201; doi:10.1186/s12859-021-04468-y)
Supplement: Supplementary file 2 — Additional file 2: Table S1. Hamming distance between hybrid and other models. [file 12859_2021_4468_MOESM2_ESM.docx]

Table S1. Hamming distance between hybrid and other models.

| ≥ 20% | Random Forest | SVC (RBF) | SVC (polynomial) | LinearSVC | KNN |
| --- | --- | --- | --- | --- | --- |
| Before Feature Selection | 0.0583 | 0.0336 | 0.0477 | 0.0795 | 0.1007 |
| After Feature Selection | 0.0777 | 0.0247 | 0.0353 | 0.0883 | 0.1431 |
