# Supplementary material for: Prediction of antimicrobial peptides toxicity based on their physico-chemical properties using machine learning techniques
Source: BMC Bioinformatics. 2021 Nov 10;22:549. doi: 10.1186/s12859-021-04468-y (PMC8582201; doi:10.1186/s12859-021-04468-y)
Supplement: Supplementary file 3 — Additional file 3: Table S2. Distribution of final features in each feature category and sub-category. [file 12859_2021_4468_MOESM3_ESM.docx]

| **Feature Category** | **Feature Sub-category** | **No.** |
| --- | --- | --- |
| Physico-chemical | Aggregation propensity in vivo | 1 |
|  | Charge density | 1 |
|  | Iso electric point | 1 |
|  | Physicochemical composition | 5 |
|  | Physicochemical distribution | 12 |
|  | Physicochemical transition | 7 |
| Autocorrelation | Geary autocorrelation | 15 |
|  | Moran autocorrelation | 16 |
|  | Normalized Moreau–Broto autocorrelation | 20 |
| Pseudo-amino acid composition | Pseudo amino acid composition I | 1 |
| Sequence order | Quasi-sequence-order | 6 |
|  | Sequence order coupling number | 3 |
| Amino acid composition | Amino acid composition | 2 |
| Total | | 90 |

Table S2. Distribution of final features in each feature category and sub-category
