# Supplementary material for: Prediction of antimicrobial peptides toxicity based on their physico-chemical properties using machine learning techniques
Source: BMC Bioinformatics. 2021 Nov 10;22:549. doi: 10.1186/s12859-021-04468-y (PMC8582201; doi:10.1186/s12859-021-04468-y)
Supplement: Supplementary file 4 — Additional file 4: Table S3. Final features sorted by their importance. [file 12859_2021_4468_MOESM4_ESM.docx]

Table S3. Final features sorted by their importance

| **Name** | **Category** | **Sub Category** |
| --- | --- | --- |
| aggregationPropensityInVivo | Physico-chemical | - |
| _PolarityC1 | Physico-chemical | Physicochemical composition |
| chargeDensity | Physico-chemical | - |
| taugrant6 | Sequence order | Sequence order coupling number |
| MoreauBrotoAuto_Hydrophobicity1 | Autocorrelation | Normalized Moreau–Broto autocorrelation |
| MoreauBrotoAuto_FreeEnergy5 | Autocorrelation | Normalized Moreau–Broto autocorrelation |
| _SecondaryStrD2001 | Physico-chemical | Physicochemical distribution |
| GearyAuto_Polarizability3 | Autocorrelation | Geary autocorrelation |
| GearyAuto_Polarizability8 | Autocorrelation | Geary autocorrelation |
| isoElP | Physico-chemical | - |
| _PolarityT23 | Physico-chemical | Physicochemical transition |
| QSOSW12 | Sequence order | Quasi-sequence-order |
| QSOgrant21 | Sequence order | Quasi-sequence-order |
| GearyAuto_ResidueVol6 | Autocorrelation | Geary autocorrelation |
| _PolarityC3 | Physico-chemical | Physicochemical composition |
| tausw8 | Sequence order | Sequence order coupling number |
| APAAC2 | Pseudo-amino acid composition | Pseudo amino acid composition I |
| MoreauBrotoAuto_Steric17 | Autocorrelation | Normalized Moreau–Broto autocorrelation |
| GearyAuto_FreeEnergy12 | Autocorrelation | Geary autocorrelation |
| QSOSW29 | Sequence order | Quasi-sequence-order |
| tausw2 | Sequence order | Sequence order coupling number |
| GearyAuto_FreeEnergy8 | Autocorrelation | Geary autocorrelation |
| _HydrophobicityD3001 | Physico-chemical | Physicochemical distribution |
| L | Amino acid composition | Amino acid composition |
| MoranAuto_AvFlexibility7 | Autocorrelation | Moran autocorrelation |
| GearyAuto_Mutability9 | Autocorrelation | Geary autocorrelation |
| _NormalizedVDWVC3 | Physico-chemical | Physicochemical composition |
| _SolventAccessibilityD1075 | Physico-chemical | Physicochemical distribution |
| MoranAuto_FreeEnergy6 | Autocorrelation | Moran autocorrelation |
| GearyAuto_AvFlexibility4 | Autocorrelation | Geary autocorrelation |
| MoreauBrotoAuto_Mutability1 | Autocorrelation | Normalized Moreau–Broto autocorrelation |
| MoranAuto_Mutability7 | Autocorrelation | Moran autocorrelation |
| MoranAuto_Polarizability7 | Autocorrelation | Moran autocorrelation |
| _PolarizabilityC2 | Physico-chemical | Physicochemical composition |
| MoreauBrotoAuto_AvFlexibility4 | Autocorrelation | Normalized Moreau–Broto autocorrelation |
| MoreauBrotoAuto_Steric1 | Autocorrelation | Normalized Moreau–Broto autocorrelation |
| _NormalizedVDWVD2001 | Physico-chemical | Physicochemical distribution |
| MoranAuto_ResidueVol10 | Autocorrelation | Moran autocorrelation |
| QSOgrant30 | Sequence order | Quasi-sequence-order |
| MoranAuto_Steric10 | Autocorrelation | Moran autocorrelation |
| MoreauBrotoAuto_Steric10 | Autocorrelation | Normalized Moreau–Broto autocorrelation |
| _PolarizabilityD2001 | Physico-chemical | Physicochemical distribution |
| MoranAuto_Hydrophobicity10 | Autocorrelation | Moran autocorrelation |
| MoreauBrotoAuto_AvFlexibility6 | Autocorrelation | Normalized Moreau–Broto autocorrelation |
| GearyAuto_ResidueASA7 | Autocorrelation | Geary autocorrelation |
| QSOgrant22 | Sequence order | Quasi-sequence-order |
| MoreauBrotoAuto_ResidueVol2 | Autocorrelation | Normalized Moreau–Broto autocorrelation |
| MoranAuto_AvFlexibility3 | Autocorrelation | Moran autocorrelation |
| MoranAuto_Steric8 | Autocorrelation | Moran autocorrelation |
| _PolarizabilityD2050 | Physico-chemical | Physicochemical distribution |
| GearyAuto_Mutability10 | Autocorrelation | Geary autocorrelation |
| GearyAuto_ResidueVol12 | Autocorrelation | Geary autocorrelation |
| MoreauBrotoAuto_Hydrophobicity12 | Autocorrelation | Normalized Moreau–Broto autocorrelation |
| MoreauBrotoAuto_Polarizability14 | Autocorrelation | Normalized Moreau–Broto autocorrelation |
| MoreauBrotoAuto_Steric11 | Autocorrelation | Normalized Moreau–Broto autocorrelation |
| GearyAuto_Mutability7 | Autocorrelation | Geary autocorrelation |
| _PolarityD3001 | Physico-chemical | Physicochemical distribution |
| MoranAuto_Mutability12 | Autocorrelation | Moran autocorrelation |
| GearyAuto_Polarizability1 | Autocorrelation | Geary autocorrelation |
| QSOSW16 | Sequence order | Quasi-sequence-order |
| MoreauBrotoAuto_ResidueVol10 | Autocorrelation | Normalized Moreau–Broto autocorrelation |
| MoranAuto_Polarizability2 | Autocorrelation | Moran autocorrelation |
| _SecondaryStrD1050 | Physico-chemical | Physicochemical distribution |
| MoreauBrotoAuto_FreeEnergy16 | Autocorrelation | Normalized Moreau–Broto autocorrelation |
| _HydrophobicityT12 | Physico-chemical | Physicochemical transition |
| GearyAuto_Steric8 | Autocorrelation | Geary autocorrelation |
| MoreauBrotoAuto_Hydrophobicity17 | Autocorrelation | Normalized Moreau–Broto autocorrelation |
| GearyAuto_Steric11 | Autocorrelation | Geary autocorrelation |
| MoranAuto_Hydrophobicity17 | Autocorrelation | Moran autocorrelation |
| MoranAuto_FreeEnergy11 | Autocorrelation | Moran autocorrelation |
| MoreauBrotoAuto_FreeEnergy10 | Autocorrelation | Normalized Moreau–Broto autocorrelation |
| MoreauBrotoAuto_FreeEnergy11 | Autocorrelation | Normalized Moreau–Broto autocorrelation |
| _ChargeD1001 | Physico-chemical | Physicochemical distribution |
| MoranAuto_ResidueVol15 | Autocorrelation | Moran autocorrelation |
| MoranAuto_ResidueASA12 | Autocorrelation | Moran autocorrelation |
| GearyAuto_Hydrophobicity10 | Autocorrelation | Geary autocorrelation |
| MoreauBrotoAuto_Hydrophobicity14 | Autocorrelation | Normalized Moreau–Broto autocorrelation |
| MoreauBrotoAuto_FreeEnergy13 | Autocorrelation | Normalized Moreau–Broto autocorrelation |
| MoreauBrotoAuto_FreeEnergy12 | Autocorrelation | Normalized Moreau–Broto autocorrelation |
| _SecondaryStrD1100 | Physico-chemical | Physicochemical distribution |
| _ChargeT12 | Physico-chemical | Physicochemical transition |
| _PolarizabilityT23 | Physico-chemical | Physicochemical transition |
| MoranAuto_AvFlexibility15 | Autocorrelation | Moran autocorrelation |
| _NormalizedVDWVD1075 | Physico-chemical | Physicochemical distribution |
| _HydrophobicityD2075 | Physico-chemical | Physicochemical distribution |
| _SecondaryStrT13 | Physico-chemical | Physicochemical transition |
| _NormalizedVDWVT13 | Physico-chemical | Physicochemical transition |
| _HydrophobicityC2 | Physico-chemical | Physicochemical composition |
| W | Amino acid composition | Amino acid composition |
| _PolarizabilityT13 | Physico-chemical | Physicochemical transition |
