# Supplementary material for: Prediction of antimicrobial peptides toxicity based on their physico-chemical properties using machine learning techniques
Source: BMC Bioinformatics. 2021 Nov 10;22:549. doi: 10.1186/s12859-021-04468-y (PMC8582201; doi:10.1186/s12859-021-04468-y)
Supplement: Supplementary file 5 — Additional file 5: Table S4. Labeling rules for toxic and non-toxic AMPs. [file 12859_2021_4468_MOESM5_ESM.docx]

| Toxic AMPs | | Non-toxic AMPs | |
| --- | --- | --- | --- |
| Hemolysis | **Concentration** | **Hemolysis** | **Concentration** |
| ≥ 5% | ≤ 10 µM | ≤ 2% | ≥ 10 µM |
| ≥ 10% | ≤ 20 µM | ≤ 5% | ≥ 20 µM |
| ≥ 15% | ≤ 50 µM | ≤ 10% | ≥ 50 µM |
| ≥ 20% | ≤ 100 µM | ≤ 15% | ≥ 100 µM |
| ≥ 30% | ≤ 200 µM | ≤ 20% | ≥ 200 µM |
| ≥ 50% | ≤ 300 µM | ≤ 30% | ≥ 300 µM |
|  |  | ≤ 50% | ≥ 500 µM |

Table S4. Labeling rules for toxic and non-toxic AMPs
