# Supplementary material for: Prediction of antimicrobial peptides toxicity based on their physico-chemical properties using machine learning techniques
Source: BMC Bioinformatics. 2021 Nov 10;22:549. doi: 10.1186/s12859-021-04468-y (PMC8582201; doi:10.1186/s12859-021-04468-y)
Supplement: Supplementary file 6 — Additional file 6: Table S5. Feature categories calculated for each peptide. [file 12859_2021_4468_MOESM6_ESM.docx]

| Feature Category | Features | No. of Features |
| --- | --- | --- |
| Physico-chemical | Net charge | 1 |
|  | Length | 1 |
|  | Aggregation propensity *in vivo* | 1 |
|  | Aggregation propensity *in vitro* | 1 |
|  | Disordering | 1 |
|  | Charge density | 1 |
|  | Isoelectric point | 1 |
|  | Normalized hydrophobicity | 1 |
|  | Normalized hydrophobic moment | 1 |
|  | Hydrophilicity | 1 |
|  | Steric hinderance | 1 |
|  | Solvation | 1 |
|  | Hydropathy | 1 |
|  | Amphiphilicity | 1 |
| Amino acid composition | Amino acid composition | 20 |
|  | Dipeptide composition | 400 |
| Composition, transition and distribution of physico-chemical properties | Physicochemical composition | 21 |
|  | Physicochemical transition | 21 |
|  | Physicochemical distribution | 105 |
| Autocorrelation | Normalized Moreau–Broto autocorrelation | 240 |
|  | Moran autocorrelation | 240 |
|  | Geary autocorrelation | 240 |
| Sequence order | Sequence order coupling number | 90 |
|  | Quasi-sequence-order | 100 |
| Pseudo-amino acid composition | Pseudo amino acid composition I | 30 |
|  | Pseudo amino acid composition II | 20 |

Table S5. Feature categories calculated for each peptide
